# Coexistence of Three Divergent mtDNA Lineages in Northeast Asia Provides New Insights into Phylogeography of Goldfish (*Carssius auratus*)

**DOI:** 10.3390/ani10101785

**Published:** 2020-10-01

**Authors:** Lei Cheng, Cuiyun Lu, Le Wang, Chao Li, Xiaoli Yu

**Affiliations:** Heilongjiang River Fisheries Research Institute, Chinese Academy of Fishery Sciences, Harbin 150070, China; lucuiyun@hrfri.ac.cn (C.L.); wangle@hrfri.ac.cn (L.W.); lichao@hrfri.ac.cn (C.L.); yuxiaoli0311@163.com (X.Y.)

**Keywords:** *Carassius auratus*, mtDNA, phylogeography, genetic structure

## Abstract

**Simple Summary:**

Goldfish (*Carassius auratus*) is a well-known fish as food and as a pet, which is also frequently used as experimental animal. A unique mtDNA sequence was detected in a sample from our experimental station, which motivated us to study genetic constitution of goldfish in Northeast Asia. Three divergent mtDNA lineages were confirmed to coexist in this region. Two of which corresponded to the known lineages (C2 and C6), which was consistent with the zoogeographical records that there were two sympatric subspecies in Amur river basin. However, the third one (lineage C7) was largely neglected in the previous studies. Our results suggested lineage C7 had a wide distribution from Central Asia to Northeast Asia.

**Abstract:**

Goldfish (*Carassius aurautus*), which is a middle size cyprinid, widely distribute throughout Eurasia. Phylogeographic studies using mtDNA markers have revealed several divergent lineages within goldfish. In this study, mtDNA variations were determined to elucidate the phylogeographical pattern and genetic structure of goldfish in Northeast Asia. A total of 1054 individuals from Amur river basin were analyzed, which including five newly collected populations and four previously reported populations. Three distinct mtDNA lineages were identified in those samples, two of which corresponded to two known lineages C2 and C6, respectively. The third lineage referred to as C7, following six known lineages of goldfish in mainland Eurasia. AMOVA results suggested that most of the genetic variations were among lineages, rather than among populations or twice samplings. We noted that the control region (CR) and cytochrome b (*cytb*) sequences of lineage C7 have been reported in previous studies, respectively. However, the evolutionary position and distribution pattern of this lineage was not discussed in the context of the species. Our results showed that “odd” CR and “hidden” *cytb* sequences from Central Asia represent the same mtDNA lineage of goldfish. The known samples of C7 lineage were collected from Central Asia (Eastern Kazakhstan and Western Mongolia) to East Asia (Northeast China and Far East Russia), which suggested that it had a wider distribution, rather than limit in Central Asia.

## 1. Introduction

Fishes of genus *Carassius* populate a wide variety of habitats throughout Eurasia, especially in East Asia. Nowadays, three species are generally considered to be valid in this genus: *C. carassius*, *C. auratus* and *C. cuvieri* [1,2,3,4]. Crucian carp (*C. carassius*), which is native to parts of Europe and Central Asia, can be diagnosed from its congeners with the free edge of the dorsal and tail fins were convex [1,3]. Goldfish (*C. auratus*) could be found from Asia to Europe, which usually further deiminated into a few subspecies [1,2,3,4]. However, it is difficult to classify goldfish into further lower taxonomic categories, because of their highly variable morphology, wide distribution, variable ploidy levels and complex reproduction modes [1,2,3,4,5,6,7]. Japanese white crucian carp (*C. cuvieri*) was previously regarded as a subspecies of *C. aurautus* [8]. A growing number of literatures treated it as a valid species, due to its genetic independence and limited distribution [9,10,11].

Wild goldfish are distributed in many regions of Eurasia, including Japanese archipelago and other East Asia affiliated islands. Ichthyologists have recognized that the goldfish in the Eurasia continent are divergent from that in Japanese archipelago for a long time [1,2]. Goldfish in Eurasia—excluding Japan—and, in particular, China are usually divided into two subspecies (*C. auratus auratus* and *C. auratus gibelio*) [1]. Goldfish in the Japanese archipelago can be classified into several other subspecies [2]. 

Mitochondrial DNA (mtDNA) is marker of choice in phylogeography and population genetics of animals [12,13], which has been proved to be a powerful tool in genetic studies of goldfish [4,9,10,11,14,15,16,17,18]. Molecular evidences supported that goldfish in Japanese archipelago and mainland Eurasia were clustered into two distinct clades, hereafter referred to as the Japanese clade (B) and Continental clade (C) [14,15]. It was suggested that the disappearance of land bridges in the Tsushima Strait around 3.0 Ma may be responsible for the separation of Japanese clade and Continental clade of *C. auratus* [15]. Furthermore, there were several lineages identified within the two major clades, respectively [14,15].

As mentioned by Takada et al. [14], an “odd” sequence of control region (CR) identified from Kazakhstan by Sakai et al. [16], would change the tree topologies of goldfish during phylogenetic analysis. This “odd” CR sequence was excluded from their study since its phylogenetic position was thought to be extremely unstable. The CR sequence of a sample from experimental station of Heilongjiang Fisheries Research Institute, was closely related to the “odd” CR sequence from Kazakhstan. Though, control region and cytochrome b (*cytb*) gene were the two most frequently used mitochondrial markers in phylogeographic and genetic studies of goldfish, however, most studies were only based on one or the other, which made data of these studies hard to be compared or reanalyzed together. We also sequenced the *cytb* gene of the sample in our experimental station. It was unexpected that its *cytb* sequence was related to “hidden” *cytb* sequences (“*C. gibelio* II”) identified by Kalous et al. [17]. Thus, mtDNA of this sample suggested that subspecies “M” by Sakai et al. [16] and “*C. gibelio* II” by Kalous et al. [17] seem to represent the same lineage of goldfish. It is hard for us to come to this conclusion based on only a single sample. Unfortunately, both Sakai et al. [16] and Kalous et al. [17] only analyzed a limited portion of known goldfish lineages (see Table S1). After the seven lineages reported by Takada et al. [14], a few additional lineages have been identified in goldfish [15]. Additionally, the “odd” CR and “hidden” *cytb* sequences were collected from Central Asia. However, our sample was unlikely to be from Central Asia, as it was more than 3000 km away from our experimental station.

The hypothesis for the study was that the unique sample in our experimental station was unintentionally collected from Northeast Asia, where our experimental station located. By reanalysis of CR sequences of another previous study [18], we found that one of three lineages were close to the “odd” CR sequence described above. Thus, we further collected samples from the similar region followed Jiang et al [18]. The first goal of the present study was to elucidate genetic structure in Northeast Asia and find more samples of each lineages. The second goal was to test if the “odd” CR sequence of Sakai et al. [16] and “hidden” *cytb* sequences of Kalous et al. [17] represent the same lineage of goldfish. The results of our study will provide new insights into the biogeography and evolution of goldfish. We found a relatively small but not negligible amount of *C. auratus* samples in this region hold CR sequences that are close to the “odd” CR sequence from Kazakhstan. All available *cytb* sequences of these samples cluster with “hidden” *cytb* sequences of Kalous et al. [17]. Thus, an additional mitochondrial lineage, which was not included in six continental lineages (C1-C6) in mainland Eurasia, was confirmed, and thought to be widely distributed from Central Asia to Northeast Asia.

## 2. Materials and Methods 

### 2.1. Sample Collection

**Figure 1:**
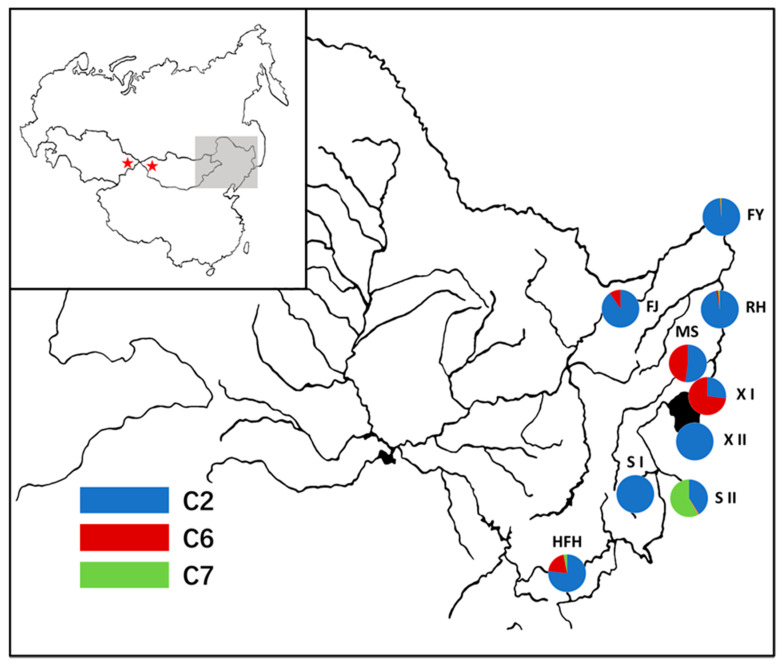
Geographical distribution and lineage constitution of each Carassius auratus population in this study. Codes for sampling localities are as follows: Fuyuan (FY); Fujin (FJ); Raohe (RH); Mishan (MS); Xingkaihu (XI); Suifenhe (SI); Huifahe (HFH); Xingkaihu in Russia (XII); Suifenhe in Russia (SII). Each lineage is uniquely colored as shown in the figure.

We collected fin clips of 668 goldfish from 5 localities of the middle reaches of the Amur river basin and surrounding areas. Control region sequences of mtDNA of Jiang et al. [18] were retrieved, because these samples were collected from a similar region and provided background information. Thus, a total of 1054 individuals from 9 populations collected from this region were analyzed in this study. Geographical distribution and lineage constitution of 9 populations were indicated in Figure 1. Fin clips were preserved in 95% ethanol. All animal procedures in this study were conducted according to the guidelines for the care and use of laboratory animals of Heilongjiang River Fisheries Research Institute, Chinese Academy of Fishery Sciences (CAFS). The studies in animals were reviewed and approved by the Committee for the Welfare and Ethics of Laboratory Animals of Heilongjiang River Fisheries Research Institute, CAFS.

### 2.2. Molecular Methods

Genomic DNA was extracted by the standard phenol-chloroform method from ethanol-fixed fin clips. PCR and sequencing primers were listed in Table 1. Control region was amplified with the primer pair L15923 [19] and Hl6500 [20] followed the previous studies. Cytochrome b gene was sequenced in a represented subset of samples in our laboratory with primer pair L14724 [21] and H15915 [21]. PCR were performed in a 30 μL volumes with 1x final concentrations of PCR mixture (Cowin bioscience, Beijing, China), 0.3 μM of each primer and ~30 ng genomic DNA. Amplifications were performed on the GeneAmp PCR system 9700 (Applied Biosystems, Foster City, CA, USA) with the following PCR profile: An initial denaturation 94 °C for 2 min, followed by 35 cycles of denaturation at 94 °C for 30 s, annealing at 60 °C for 30 s and extension at 72 °C for 1 min, followed by a final extension at 72 °C for 7 min. Primers 16500F and 15372F were designed for the sequencing control region and cytochrome b gene, respectively. After being purified, PCR products were sequenced using the ABI 3730xl sequencing system. Then, raw trace files were revised using the software Finch TV (Geospiza, Inc.). 

### 2.3. Population Genetic Analyses

DNA sequences were aligned using the MUSCLE program with default parameters [22]. DNAsp v6 [23] was used to identify unique CR haplotypes. Obtained sequence data were deposited in the GenBank database under Accession Numbers MT199236-MT199260. Number of segregating sites (S), mean number of nucleotide differences (K), Haplotype diversity (*Hd*) and Nucleotide diversity (π) and pairwise differentiation (*Φ*_ST_) between populations were calculated using Arlequin v3.5 [24]. Analysis of molecular variance (AMOVA), implemented in Arlequin, was used to estimated hierarchical structuring genetic variations. Two different partitions of datasets were applied into AMOVA. First, we determined the variation partitioned between geographical regions according to the sampling site. Since the time interval of twice samplings was about 10 years, we took them as two independent groups, which allowed us to test whether there was a significant temporal difference. Phylogenetic analysis based on mtDNA revealed that there were three distinct mtDNA lineages in this region. The second partition investigated variation between and within the three lineages. 

### 2.4. Phylogenetic Analyses

To clarify haplotype phylogenetic relationship in the context of genus *Carassius*, DNA sequences identified by a couple of the previous studies were involved in phylogenetic analyses. Detailed information about DNA sequences used in the phylogenetic analysis can be found in Supplementary Materials Table S1. Three different datasets were subjected to phylogenetic analysis: (1) control region (CR) sequences; (2) *cytb* sequences; and (3) concatenated CR and *cytb* sequences. Each dataset was analyzed using neighbor-joining (NJ), maximum likelihood (ML) and Bayesian inference (BI) method, respectively. The neighbor-joining method was implemented in the MEGA-X package [25], using the distances corrected based on the maximum composite likelihood model. Branch supports for NJ trees were measured by bootstrap analysis with 1000 random replicates. We inferred the maximum-likelihood tree by combining ModelFinder, tree search, SH-aLRT test and ultrafast bootstrap with 1000 replicates in IQ-TREE [26]. Bayesian inference was performed using MrBayes [27] under the optimized model determined by the mrModelTest [28] program according to Akaike information criterion for each dataset. Monte Carlo Markov chains run for 10,000,000 generations starting from a random tree. Trees and parameters were sampled every 100 generations. The first 25% of the trees were discarded as burn-in and the remaining trees were used to generate a consensus tree. Branch support for BI trees was based on posterior probabilities (PP). Median-joining algorithm implemented in Network 5.0 was used to reconstruct evolutionary relationships of CR haplotypes. 

## 3. Results

### 3.1. Genetic Diversity 

Out of the 357bp aligned sequences, 56 nucleotide positions were variable. Indels were observed in 24 sites, and a 18bp-indel was observed in the 5′ end of two haplotypes. Transitions and transversions occurred in 37 and 2 sites, respectively. The pattern of polymorphism indicating that different types of variation reoccurred in some loci and there was a strong transitional bias. A total of 58 unique haplotypes were identified from 1054 CR sequences. Thirty-nine of 58 haplotypes were detected from our newly collected samples and 35 were reported by Jiang et al. [18]. Thus, 16 haplotypes were shared between our samples and Jiang et al. [18]. Details of genetic diversity were summarized in Table 2. The average haplotype diversity of nine populations was 0.676 ± 0.016, with values ranged from 0.208 ± 0.054 to 0.878 ± 0.013. The average nucleotide diversity (π) of total populations was 1.798 ± 0.942(%), with values ranged from 0.396 ± 0.271(%) to 2.197 ± 1.142(%). 

### 3.2. Genetic Structure

**Figure 2:**
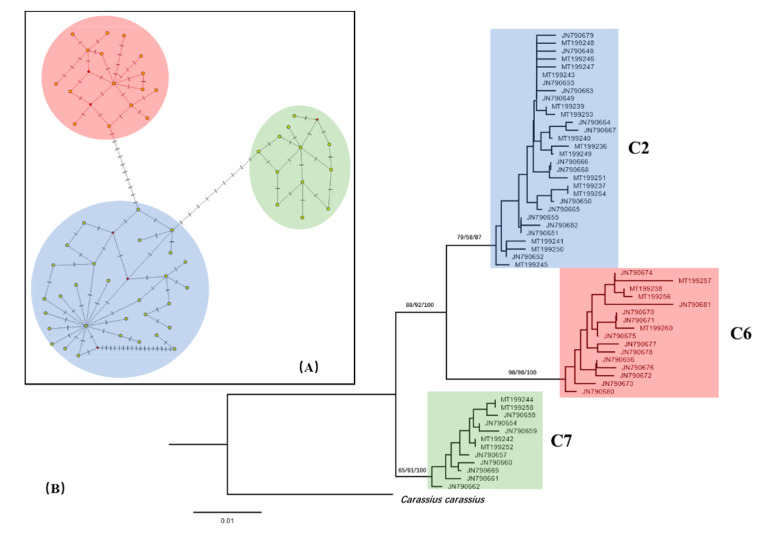
(**A**) median-joining network of 58 CR haplotypes of goldfish (*Carassius aurautus*) in Northeast Asia based on CR sequences. Each line-segment represents a single base pair change. There was a 18 bp indel in two haplotypes of lineage C2. (**B**) neighbor-joining (NJ) phylogeny of 58 CR haplotypes of goldfish (*Carassius aurautus*).

Population distribution of CR haplotypes was presented in Table 3. As shown, the most common haplotype (JN790649) was found in 55.9% samples, and the haplotype was dominant in twice samplings by us and Jiang et al. [18]. CR haplotype network showed three divergent haplotype clusters, consistent with results of phylogenetic analysis (Figure 2). The dominant haplotype (JN790649) was core of lineage C2 in this region, which was surrounded by a star-like pattern. As shown in Figure 1 and Table 3, lineages C2 and C6 account for almost half of samples in populations MS and XI, while population SII mainly consists of lineages C2 and C7. With the above three exceptions, the other populations were mainly composed of lineage C2. Population structure and geographical subdivision of goldfish in the Northeast Asia were estimated based on *Φ*_ST_ (Table 4). Pairwise *Φ*_ST_ were ranged from 0.024 (between FY and XII) to 0.656 (between FY and XI), and most populations showed significant differentiation.

AMOVA results (Table 5), indicated that the differentiations of three lineages could explained majority of the total genetic variance (88.7%). There was no significant difference between the twice samplings (*Φ*_CT_ = -0.025, *p* = 0.423 ± 0.015). This can be verified by dominant haplotype (JN790649) in twice samplings was the same (Table 3). The AMOVA results also found that a substantial proportion of molecular variance was attributable to differences among populations (*Φ*_ST_ = 0.326).

### 3.3. Phylogeny of Carassius Auratus Complex

**Figure 3:**
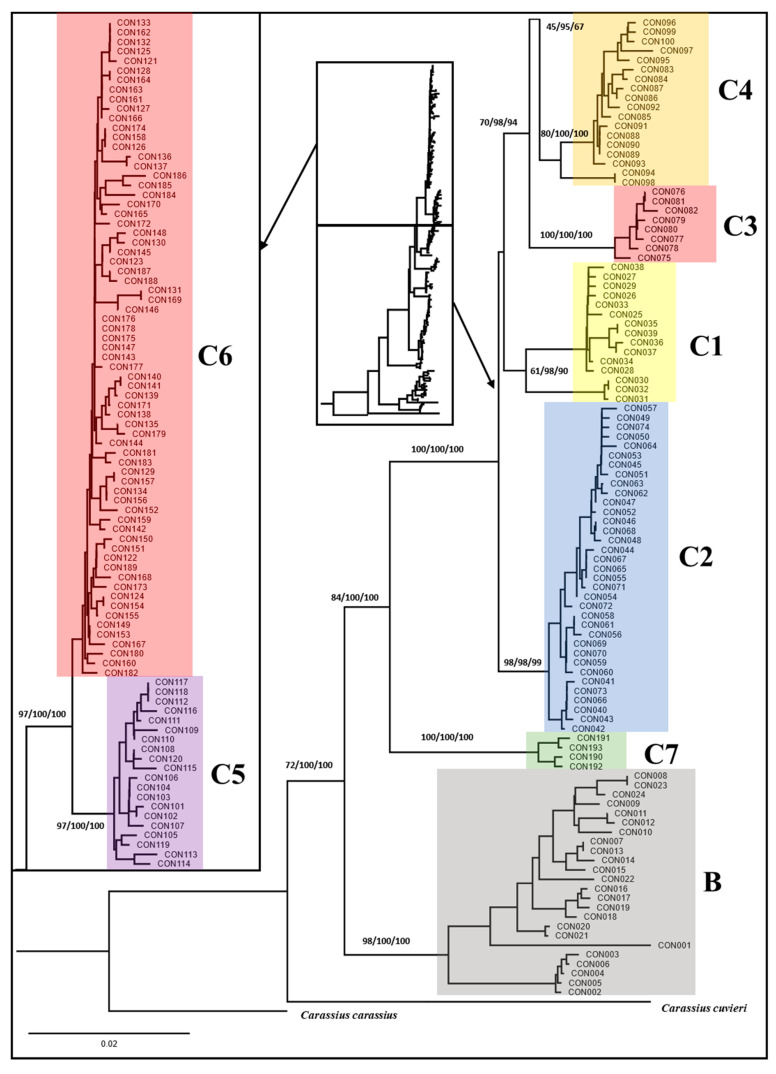
NJ phylogeny of the genus *Carassius* based on concatenated sequences of *cytb* gene and control region. As maximum likelihood (ML) method and Bayesian inference (BI) showed the same topology, only the NJ tree is presented here. Numbers near the branches represent branch support for neighbor-joining, maximum likelihood method and Bayesian inferences, respectively.

The trees generated from NJ, ML and BI analyses are highly congruent with each other for the same dataset. For concatenated dataset, all trees revealed four major clades, which corresponding to *C. carassius*, *C. cuvieri* (Clade A in Gao et al. [15]), Japanese *C. auratus* (clade B) and continental *C. auratus* (clade C). As shown in Figure 3, *C. carassius* deepest split from others clades, and *C. cuvieri* was a sister taxon to the two major clades (B and C) of *C. auratus*. Clade B, mainly including samples from the main islands of Japan and northern Ryukyus. Clade C, containing specimens from Eurasian continent, Taiwan and the south-central Ryukyus islands, which further subdivide into seven lineages. Four lineages (C2, C3, C4, C6) were identified by Takada et al. [14], and two additional lineages (C1 and C5) were reported by Gao et al. [15] The remaining lineage (C7) was defined by this study and followed the known six lineages. A total of 12 haplotypes of lineage C7 were identified in this study, including 8 haplotypes from Jiang et al. [18] and 4 haplotypes from ours. However, there was no haplotype shared between the twice samplings (Table 3). In SII population, 52 out of 90 individuals were from lineage C7, but there were only 7 individuals belonged to lineage C7 in our collection (a total of 668 individuals). All CR sequences of these 7 samples were closely related to “odd” CR of Sakai et al. [16], and their *cytb* sequences were like that of "*C. gibelio* II" [17]. One of the seven samples had identical CR and *cytb* haplotypes to those of the unique sample in our experimental station. Our results supported that “odd” CR of Sakai et al. and “hidden” *cytb* sequences of Kalous et al. represent the same lineage [16,17], which were neglected by Takada et al. [14] and further studies [15,18,29].

Trees based on the sequences of *cytb* gene corroborated the existence of distinct lineages in goldfish as revealed by the concatenated dataset. For *cytb* dataset, a remarkable difference between NJ tree and the two other trees (ML and BI) was the position of clade A (corresponding to *C. cuvieri*). In NJ tree based on *cytb* sequences, clade A is closer to the Japanese clade (B) than to the Continental clade (C), rather than a sister clade to the whole *C. auratus* as shown in NJ tree based on concatenate sequences (see Figure S1). In trees based on the CR sequences, all lineage could be retrieved, but the relationship among lineages within *C. auratus* could not be clearly resolved (see Figure S2). This discrepancy probably due to saturation which has been mentioned in previous studies [14].

## 4. Discussion

In this study, we elucidated mtDNA variations of goldfish in Northeast Asia, then revised the phylogeny of genus *Carassius*. Crucian carp (*C. carassius*) formed a deep divergent clade, which supported crucian carp is the most distinct species within this genus [1,3,4]. For all three datasets, Japanese white crucian carp (*C. cuvieri*) formed another major clade, but the phylogenetic position of this taxon is not stable. In most trees, *C. cuvieri* was a sister taxon to the whole *C. auratus*, including Japanese clade (B) and continental clade (C). In NJ tree based on *cytb* sequences (Figure S1), *C. cuvieri* is closer to the Japanese clade than the continent clade. Japanese crucian carp used to be a subspecies of *C. auratus* [8], but was regarded as a valid species now [9,10,11]. Almost all phylogenic and population studies indicated Japanese white crucian carp was a distinct taxon, but many studies based on *cytb* sequences also suggested *C. cuvieri* are genetically closer to Japanese *C. auratus* than non-Japanese ones [17,30,31]. We tend to regard Japanese white crucian carp as a valid species, but its taxonomy may need further confirm, since its differences from other groups of *Carassius auratus* are relatively small [2,8].

The samples of *C. auratus* native to Japan’s islands and North Ryukyu islands formed the clade B, which were divergent from goldfish in mainland Eurasia. In Japan, fishes of *C. auratus* have been classified into several subspecies [2,8] based on morphological and genetical criteria, such as ginbuna (*C. a. langsdorfii*), kinbuna (*C. a. subsp*), nagabuna (*C. a. burgeri*) and nigorobuna (*C. a. grandoculis*). Though distinct lineages can be further detected in Japanese clade, no exact match between the above subspecies and lineage was confirmed in Japanese clade [9,10,11,14]. The classification criteria among these subspecies are very vague. For example, Japanese ichthyologists tend to take gynogenetic reproduction and polyploidy as diagnostic characteristics of *C. a. langsdorfii* [2]. However, previous studies have revealed that gynogenetic polyploids in all three known lineages within Japanese clade, and a sizable chunk of haplotypes were shared between polyploids and diploids [14,15,29]. 

In Continent clade, multiple mitochondrial lineages, which were associated to geographical distribution, have also been identified. Ichthyologists traditionally divided *C. auratus* in Eurasia into two subspecies: *C. auratus auratus* and *C. auratus gibelio* [1,3]. Among continental clade, lineages C2 and C6 are the most widely distributed ones, and thought to be associated with above two subspecies, respectively [14,15,16,17,32]. Subspecies *C. auratus auratus* is naturally distributed in all parts of China outside the Qinghai Tibet Plateau [1]. Besides lineage C6, there were a couple of cryptic lineages have been found within the native range of *C. auratus auratus*. Gao et al. have identified lineage C5 distributed in the middle and lower reaches of the Yangtze River, and lineage C1 distributed in Fujian, Vietnam [15]. Meanwhile. The majority of *C. auratus* from south Ryukyu Islands (lineage C4) and Taiwan island (lineage C3) also belong to the continental superclade [15]. Subspecies *C. auratus gibelio,* thought to be widespread, at least from central Europe to East Asia, but exact limits are not clear [3,33]. The native distribution of *C. auratus gibelio* in China is limited to the Amur river basin and the Irtysh river basin [1]. Consistent with Jiang et al. [18], three distinct mitochondrial lineages were also detected in newly sampled individuals in this study. According to zoogeographical records, the Northeast Asia is an overlapping range of the *C. auratus auratus* and *C. auratus gibelio* [1]. Among three mitochondrial lineages we identified, two lineages just corresponded to *C. auratus auratus* (lineage C6) and *C. auratus gibelio* (lineage C2), respectively. However, the third lineage was clustered with the “odd” CR detected by Saikai et al. [16]. We sequenced the cytochrome B gene of the corresponding samples and found that they exclusively belonged to “*C. gibelio* II” reported by Kalous et al. [17]. These results suggested that subspecies “M” of Saikai et al. [16] and “*C. gibelio* II” of Kalous et al. [17] represent the same mtDNA lineage. We referred to this new lineage as C7 followed the order of Gao et al, which was not included in the phylogenetic trees constructed by Gao et al. [15] 

As mentioned above, our samples were collected from Northeast Asia (Amur river basin), but samples of lineage C7 were also found in Central Asia (Kazakhstan, Mongolia) [16,17]. The localities of the samples suggested that lineage C7 seems to be widely distribute across mid-latitude Asia from central to northeast. Thus, the distribution of lineage C7 is high overlapped with that of lineage C2. The first description of *C. auratus gibelio* by Bloch (1782) was based on samples collected from “Churmark, Pommern, Schlesien und Preussen” (historical areas of eastern and central Europe) [17]. However, it has also been proposed that *C. auratus gibelio* was introduced from East Asian, rather than native to Europe [16,33]. Sakai et al. suggested that subspecies “M” (lineage C7) seem to be a native form, probably the same fish that was recorded as *C. auratus gibelio* by Bloch (1782) [16]. Unfortunately, the type specimens *C. auratus gibelio* has been lost, and the original syntype has been replaced by a specimen of *C. carassius* during former investigations [17]. A specimen ZMB 33979 was designate as neotype of *C. auratus gibelio*, because the neotype comes from part of the type locality and corresponds in all investigated morphologic characters to the description given by Bloch (1782). However, mtDNA of the neotype belong to “Europe-China clade of *C. gibelio*” (lineage C2) rather than the “Mongolian clade” (lineage C7) [17]. One hypothesis is that lineage C2 originated from the east side of the Mongolian Plateau and C7 originated from west side, then range expansion or artificial introductions of lineages C2 and C7 led to the present pattern. The above scenario or other assumptions may be true, but the currently available data is not enough to draw a conclusion.

## 5. Conclusions

A unique mtDNA from a sample of our experimental station promoted us to investigated the genetic diversity and phylogeny of goldfish in Northeast Asia. Our results confirmed that there were three divergent mitochondrial lineages in this region. Two of which correspond to the known lineages C2 and C6, respectively. The remaining third lineage posed CR sequences close to that of Sakai et al. Our results suggested the “odd” CR sequences by Sakai et al. [17] and “hidden” *cytb* sequences by Kalous et al. [18] were from the same lineage of goldfish. This lineage was referred to as C7 followed six known lineages in mainland Eurasia. Considering the distribution of sampling sites, the C7 lineage is likely to widely distribute from Central Asia to Northeast Asia.

## Figures and Tables

**Table 1:** PCR and sequencing primers used in the present study.

Target	Primer	Sequence (5′-3′)	References
control region	L15923	TTAAAGCATCGGTCTTGTAA	[19]
	Hl6500	GCCCTGAAATAGGAACCAGA	[20]
	16500F	AGCGCCCAGAAAAGAGAGAT	This study
cytochrome b gene	L14724	GACTTGAAAAACCACCGTTG	[21]
	H15915	CTCCGATCTCCGGATTACAAGAC	[21]
	15372F	GACCTACCCACACCATCCAA	This study

**Table 2:** Sampling information and basic genetic diversity indices of each population or lineage of *Carassius auratus*.

Population	Code	*n*	*h*	*S*	*K*	H*d*	*π* (%)
Fujin, China	FJ	135	15	38	4.191 ± 2.095	0.673 ± 0.042	1.174 ± 0.650
Fuyuan, China	FY	188	15	31	1.430 ± 0.876	0.466 ± 0.044	0.399 ± 0.271
Mishan, China	MS	119	8	22	7.263 ± 3.426	0.591 ± 0.024	2.035 ± 1.063
Raohe, China	RH	127	16	43	4.065 ± 2.041	0.602 ± 0.050	1.139 ± 0.633
Huifahe, China	HFH	99	14	46	6.908 ± 3.277	0.689 ± 0.047	1.935 ± 1.017
Suifenhe, China	SI	100	7	21	1.942 ± 1.112	0.208 ± 0.054	0.544 ± 0.345
Suifenhe, Russia	SII	90	11	24	7.512 ± 3.541	0.780 ± 0.023	2.104 ± 1.099
Xingkaihu, China	XI	100	15	24	7.842 ± 3.680	0.878 ± 0.013	2.197 ± 1.142
Xingkaihu, Russia	XII	96	13	28	1.418 ± 0.875	0.563 ± 0.057	0.396 ± 0.271
Lineage	C2	822	30	38	1.564 ± 0.934	0.479 ± 0.022	0.439 ± 0.290
	C6	173	16	18	2.327 ± 1.279	0.756 ± 0.029	0.657 ± 0.400
	C7	59	12	8	1.193 ± 0.775	0.679 ± 0.056	0.336 ± 0.242
Total		1054	58	57	6.417 ± 3.042	0.676 ± 0.016	1.798 ± 0.942

*n*: Number of individuals; *h*: Number of Haplotypes; *S*: Number of segregating sites; *K*: Mean number of nucleotide differences; *Hd*: Haplotype diversity; and π: Nucleotide diversity.

**Table 3:** The population and lineage distributions of control region (CR) haplotypes in 9 populations of goldfish (*Carassius auratus*).

Lineage	GenBank NO.	FJ	FY	MS	RH	HFH	SI	SII	XI	XII
C2	JN790648	5	5			7				13
	JN790649	74	136	56	79	53	89	22	18	62
	JN790650	5	18	1	4		2			4
	JN790651									1
	JN790652	6	9	3	6	7			1	1
	JN790653		2		1					1
	JN790655		4		7			15	1	1
	JN790663									1
	JN790664									6
	JN790665				6					2
	JN790666	20	4		3		1		2	2
	JN790667									1
	JN790668	2								1
	JN790679								5	
	JN790682			1			2			
	MT199236	2		1						
	MT199237	5								
	MT199239	1			9	2				
	MT199240	2								
	MT199241		2							
	MT199243		2		2					
	MT199245		1							
	MT199246		1							
	MT199247		1							
	MT199248				1					
	MT199249				1					
	MT199250				1					
	MT199251				2					
	MT199253				1					
	MT199254					7				
C6	JN790656	6		52	2	12	3	1	3	
	JN790670	2							16	
	JN790671			1					17	
	JN790672								17	
	JN790673								8	
	JN790674	3	1	4		2			1	
	JN790675								6	
	JN790676	1				1			1	
	JN790677								1	
	JN790678					2			3	
	JN790680						1			
	JN790681						2			
	MT199238	1								
	MT199256					1				
	MT199257					1				
	MT199260					1				
C7	JN790654							31		
	JN790657							3		
	JN790658							13		
	JN790659							1		
	JN790660							1		
	JN790661							1		
	JN790662							1		
	JN790669							1		
	MT199242		1							
	MT199244		1			1				
	MT199252				2					
	MT199258					2				
Total		135	188	119	127	99	100	90	100	96

**Table 4:** Pairwise *Φ*_ST_ (below diagonal) and associated *p* values (above diagonal) between populations of goldfish (*Carassius auratus*).

Population Code	FJ	FY	MS	RH	HFH	SI	SII	XI	XII
FJ		0.000 ± 0.000	0.000 ± 0.000	0.001 ±0.001	0.004 ± 0.002	0.014 ± 0.003	0.000 ± 0.000	0.000 ± 0.000	0.000 ± 0.000
FY	0.060		0.000 ± 0.000	0.000 ± 0.000	0.000 ± 0.000	0.005 ± 0.003	0.000 ± 0.000	0.000 ± 0.000	0.002 ± 0.001
MS	0.245	0.442		0.000 ± 0.000	0.000 ± 0.000	0.000 ± 0.000	0.000 ± 0.000	0.000 ± 0.000	0.000 ± 0.000
RH	0.034	0.032	0.335		0.000 ± 0.000	0.002 ± 0.001	0.000 ± 0.000	0.000 ± 0.000	0.007 ± 0.003
HFH	0.028	0.150	0.110	0.092		0.000 ± 0.000	0.000 ± 0.000	0.000 ± 0.000	0.000 ± 0.000
SI	0.026	0.027	0.338	0.029	0.089		0.000 ± 0.000	0.000 ± 0.000	0.003 ± 0.002
SII	0.405	0.558	0.346	0.434	0.296	0.484		0.000 ± 0.000	0.000 ± 0.000
XI	0.475	0.656	0.139	0.540	0.326	0.563	0.436		0.000 ± 0.000
XII	0.059	0.024	0.398	0.029	0.138	0.029	0.510	0.605	

**Table 5:** Analysis of molecular variance (AMOVA) among nine populations of goldfish (*Carassius auratus*).

Partitions	Source of Variation	d.f.	Sum of Squares	Variance Components	Percentage of Variation	Fixation Indices	*p*-Value
I							
	Among groups	1	92.32	−0.083	−2.51	*Φ*_CT_ = −0.025	0.423 ± 0.015
	Among populations within groups	7	962.80	1.159	35.13	*Φ*_SC_ = 0.343	0.000 ± 0.000
	Within populations	1045	2323.58	2.224	67.38	*Φ*_ST_ = 0.326	0.000 ± 0.000
	Total	1053	3378.70	3.300			
II							
	Among lineages	2	2502.10	6.559	88.72	*Φ*_ST_ = 0.887	0.000 ± 0.000
	Within lineages	1051	876.60	0.834	11.28		
	Total	1053	3378.70	7.393			

## Supplementary Materials

The following are available online at https://www.mdpi.com/2076-2615/10/10/1785/s1, Table S1: DNA sequences of control region and cytochrome b gene used in phylogenetic analysis in this study, Figure S1: Phylogeny of the genus *Carassius* based on sequences of *cytb* gene, Figure S2: Phylogeny of the genus *Carassius* based on sequences of control region.
