# Supplementary material for: Coexistence of Three Divergent mtDNA Lineages in Northeast Asia Provides New Insights into Phylogeography of Goldfish (Carssius auratus)
Source: Animals (Basel). 2020 Oct 1;10(10):1785. doi: 10.3390/ani10101785 (PMC7650769; doi:10.3390/ani10101785)
Supplement: Supplementary file 1 [file animals-10-01785-s001.zip › animmals_appendix_proofreading/animals-926669_supplementary figures.docx]

Supplementary Materials: Coexistence of Three Divergent mtDNA Lineages in Northeast Asia Provides New Insights into Phylogeography of Goldfish (*Carssius auratus*)

Lei Cheng*, Cuiyun Lu, Le Wang, Chao Li and Xiaoli Yu

Heilongjiang River Fisheries Research Institute, Chinese Academy of Fishery Sciences, Harbin 150070, China; [lucuiyun@hrfri.ac.cn](javascript:void(0);) (C.L.); [wangle@hrfri.ac.cn (L.W.)](mailto:lucuiyun@hrfri.ac.cn%20(L.W.)); [lichao@hrfri.ac.cn (C.L.)](mailto:lichao@hrfri.ac.cn%20(C.L.)); [yuxiaoli0311@163.com](javascript:void(0);) (X.Y.)

***** Correspondence: [chenglei@hrfri.ac.cn](javascript:void(0);)

Received: 25 August 2020; Accepted: 19 September 2020; Published: date

**Figure S1.** Neighbor-joining (NJ) phylogeny of the genus *Carassius* based on sequences of *cytb* gene. Maximum likelihood (ML) and Bayesian inference (BI) were high similar the NJ tree presented here, but clade A is closer to the Japanese clade (B) than to the Continental clade (C), rather than a sister clade to the whole *C. auratus* as shown in other trees.

**Figure S2.** Neighbor-joining (NJ) phylogeny of the genus *Carassius* based on sequences of control region. In trees based on the CR sequences including ML and BI methods, all lineage could be retrieved, but the relationship among lineages within *C. auratus* couldn’t be clearly resolved. This discrepancy probably due to saturation which has been mentioned in previous studies.
